# Depression Detection and Diagnosis Based on Electroencephalogram (EEG) Analysis: A Systematic Review

**DOI:** 10.3390/diagnostics15020210

**Published:** 2025-01-17

**Authors:** Kholoud Elnaggar, Mostafa M. El-Gayar, Mohammed Elmogy

**Affiliations:** 1 Information Technology Department, Faculty of Computers and Information, Mansoura University, Mansoura 35516, Egypt; kholoudamer@mans.edu.eg; 2 Department of Computer Science, Arab East Colleges, Riyadh 11583, Saudi Arabia

**Keywords:** mild depression disorder (MDD) detection, EEG signal features and biomarkers, optimizing electroencephalogram (EEG) channel selection, EEG preprocessing methods, integrating IoT and EEG, ML and DL methods for depression diagnosis

## Abstract

**Background:** Mental disorders are disturbances of brain functions that cause cognitive, affective, volitional, and behavioral functions to be disrupted to varying degrees. One of these disorders is depression, a significant factor contributing to the increase in suicide cases worldwide. Consequently, depression has become a significant public health issue globally. Electroencephalogram (EEG) data can be utilized to diagnose mild depression disorder (MDD), offering valuable insights into the pathophysiological mechanisms underlying mental disorders and enhancing the understanding of MDD. **Methods:** This survey emphasizes the critical role of EEG in advancing artificial intelligence (AI)-driven approaches for depression diagnosis. By focusing on studies that integrate EEG with machine learning (ML) and deep learning (DL) techniques, we systematically analyze methods utilizing EEG signals to identify depression biomarkers. The survey highlights advancements in EEG preprocessing, feature extraction, and model development, showcasing how these approaches enhance the diagnostic precision, scalability, and automation of depression detection. **Results:** This survey is distinguished from prior reviews by addressing their limitations and providing researchers with valuable insights for future studies. It offers a comprehensive comparison of ML and DL approaches utilizing EEG and an overview of the five key steps in depression detection. The survey also presents existing datasets for depression diagnosis and critically analyzes their limitations. Furthermore, it explores future directions and challenges, such as enhancing diagnostic robustness with data augmentation techniques and optimizing EEG channel selection for improved accuracy. The potential of transfer learning and encoder-decoder architectures to leverage pre-trained models and enhance diagnostic performance is also discussed. Advancements in feature extraction methods for automated depression diagnosis are highlighted as avenues for improving ML and DL model performance. Additionally, integrating Internet of Things (IoT) devices with EEG for continuous mental health monitoring and distinguishing between different types of depression are identified as critical research areas. Finally, the review emphasizes improving the reliability and predictability of computational intelligence-based models to advance depression diagnosis. **Conclusions:** This study will serve as a well-organized and helpful reference for researchers working on detecting depression using EEG signals and provide insights into the future directions outlined above, guiding further advancements in the field.

## 1. Introduction

In modern society, numerous individuals encounter obstacles in meeting their job responsibilities within the given timeframe. This has led to an increase in cases of anxiety and depression globally [1]. Due to population growth, mental health issues are becoming more common in many nations, both developed and developing [2]. The COVID-19 pandemic has exacerbated the situation further [3]. Per the World Health Organization (WHO), depression is among the most prevalent mental illness and is the second leading cause. It affects around 320 million people worldwide [4]. According to statistics, the number of people who are experiencing depression has increased by 18.0% in the last decade [5], with female patients outnumbering males by a wide margin. Those who have mild depression may have unpleasant feelings, trouble sleeping, and a lack of enthusiasm for doing things, while those with severe depression may exhibit suicidal tendencies.

Depression has the potential to threaten the welfare of billions of families [6]. The American Psychiatric Association produced the DSM-V [7], which is currently the most widely accepted international standard for diagnosing depression. The Chinese Classification of Mental Disorders, Third Edition (CCSD-III) is the most widely used Chinese standard for diagnosing depression in China. These diagnostic techniques use scores developed through in-person interviews with sad people and their relatives to ascertain whether the patient exhibits symptoms of depression.

Consequently, the present approach is subjective and relies on the doctors’ expertise, which can be time-consuming and prone to mistakes. The proportion of mental health patients seeing psychiatrists or therapists has decreased even more. In light of this crisis, researchers and scientists worldwide have endeavored to create alternative solutions and completely automated methods for monitoring and detecting depression [8].

Depression can be detected using many features and indicators [9]. The first feature is a textual-based feature approach that uses social media texts to predict depression [10]. The second is a real-time acoustic-based feature that uses speech analysis to detect depression [11]. The third is a facial expression and eye movement-based feature that detects depression using video analysis [12], and finally, an electroencephalography (EEG)-based feature.

**Figure 1:**
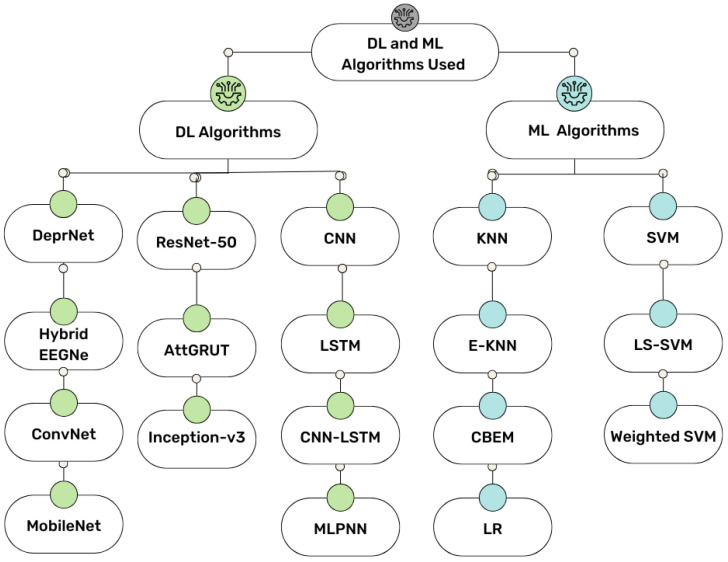
The diversity of DL and ML algorithms used in prior studies for EEG-based depression detection.

For detecting depression, machine learning (ML) and deep learning (DL) techniques should adopt an information flow inspired by the diagnostic process employed by clinicians, as illustrated in Figure 1 [13]. This approach incorporates multiple communication types, each contributing unique insights into depression detection [14]. These communication types include the following:

- **Visual Indicators:** Body movements, facial expressions, and muscle activity are commonly studied for depression detection, but their interpretation is subjective and prone to observer bias [15].
- **Speech Indicators:** Acoustic features, such as tone, pitch, and rhythm, can reflect cognitive or physiological changes linked to depression, though cultural and personal variations introduce subjectivity [11].
- **Text indicators:** Written language analysis explores linguistic patterns and non-semantic features like syntax and word frequency, but its interpretation remains subjective, particularly across diverse populations [16,17].
- **Biological Indicators:** Biological signals, especially EEG and eye movement tracking, offer objective and reproducible measures of depression. EEG, in particular, provides direct insights into brain activity, minimizing bias and delivering reliable data for diagnosis [18].

**Focus on EEG**

While visual, speech, and text indicators enrich the understanding of depression through multi-modal data integration, EEG signals hold a distinct advantage due to their objectivity and direct connection to brain activity. This survey, therefore, emphasizes EEG-based methods, leveraging their ability to provide robust and unbiased inputs for ML and DL models in depression diagnosis. This review highlights the potential for developing highly accurate and scalable computational tools for mental health applications by focusing on EEG.

In recent studies [5,19,20,21,22], EEG data have been investigated as a suitable diagnostic method for the detection of the neurological disorder. This is non-invasive and less expensive than other methods. With a time reference, EEG signals record data about the brain’s central nervous system and brain activities [23]. Postsynaptic potentials in cerebral cortical neurons comprise the EEG, the total of these potentials [24]. The technology for EEG acquisition offers non-camouflage and real-time differences. Commonly, it is employed to assess the functional state of the brain in a clinical context. A sensor can gather the EEG voltage produced in the brain through cortical conduction.

Due to their intricacy, observable abnormalities would be challenging to find with the naked eye. Despite their difficulties, these properties have led to the recognition of physiological signals as valuable tools for depression detection. Several studies have shown that EEG signals can tell the difference between normal and depressed patients [25]. The change rates in the power, band, amplitude, and other variables in healthy and depressed individuals’ EEGs differ [26]. EEG data can be analyzed using data mining methods to identify depression-related attributes. In this domain, various ML and DL techniques, as well as various feature selection methods, are growing in popularity. The following points outline the key contributions of this survey:

- **Classification Methods:**
  The survey provides a comprehensive review of various classification methods for diagnosing depression based on EEG signals, particularly on ML and advanced DL techniques.
- **EEG Datasets:** Different EEG datasets from local and public sources are gathered and analyzed to ensure a broad and representative evaluation.
- **Preprocessing Techniques:** The survey details different techniques for preprocessing EEG data, including filters and methods for removing artifacts, to ensure data quality and accuracy.
- **Feature Extraction Approaches:** Approaches for extracting features from various categories, such as spectral, non-linear, spatial, statistical, and wavelet transform, are thoroughly reviewed and compared.
- **Future Directions and Challenges:** The survey addresses future directions and challenges in enhancing depression diagnosis, including the following:
  – **Data Augmentation:** Techniques to improve the robustness of models through data augmentation. EEG Channel Selection: Strategies for optimizing EEG channel selection for more accurate depression diagnosis.
  – **Transfer Learning and Encoder-Decoder Architectures:** Opportunities to leverage pre-trained models and improve diagnostic accuracy through transfer learning and encoder-decoder architectures using deep neural networks.
  – **Feature Extraction Techniques:** Investigating new feature extraction techniques to enhance the performance of ML and DL models for automated depression diagnosis.
  – **IoT Integration:** Exploring the integration of Internet of Things (IoT) devices with EEG for remote patient monitoring, facilitating continuous and real-time mental health assessment.
  – **Distinguishing Depression Types:** Research focused on distinguishing between different types of depression, a critical area for improving diagnostic precision.
  – **Comprehensive Reference:** This survey will serve as a well-organized and helpful reference for researchers working on detecting depression using EEG signals, providing insights into the outlined future directions and guiding further advancements in the field.

**Figure 2:**
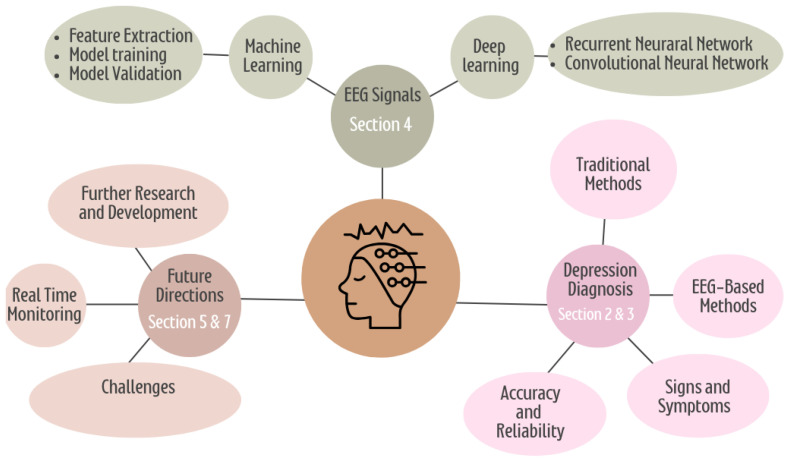
The detailed mapping of interconnections between survey sections.

The survey is organized as follows. Section 2 describes the methodology for detecting depression. Section 3 details the five significant steps of the depression diagnosis pipeline. Section 4 reviews the literature based on the ML and DL approaches. Section 5 illustrates the discussion and comparison analysis. Section 6 explains the EEG datasets employed for diagnosing or predicting the treatment outcomes of depression. Section 7 discusses the prospective directions for this research. Lastly, in Section 8, we present the conclusion of the study. The entity relationship between the sections is illustrated in Figure 2.

## 2. Methods

**Figure 3:**
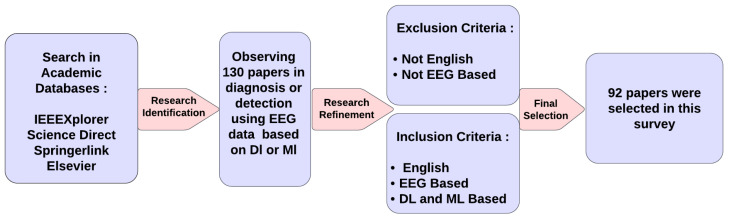
The paper selection methodology flow chart.

Aligned with the PRISMA guidelines [27], this section systematically reviews and synthesizes the methodologies explored for depression detection since 2016, providing a transparent and comprehensive overview of existing approaches. The search terms, sources of data, criteria for inclusion and exclusion, and criteria for selecting articles are all covered. Figure 3 shows the summary of this methodology. Frequency-based analyses of the methods and sub-methods utilized for detecting depression are presented in Table 1 and Table 2.

### 2.1. Research Question

This review aims to address several critical research questions to better understand the current landscape of EEG-based depression diagnosis using ML and DL approaches. The following questions guide the exploration of existing studies:

- **AQ1:** What ML and DL models are most effective in classifying EEG signals for depression diagnosis?
- **AQ2:** How do various EEG preprocessing methods impact the performance of ML and DL models in depression detection?
- **AQ3:** What are the primary EEG signal features and biomarkers for depression detection?
- **AQ4:** What challenges and research gaps exist in using EEG for depression detection and diagnosis?

### 2.2. Search Keywords

First, a search was conducted using carefully chosen keywords. After performing a preliminary search, additional words discovered in pertinent articles were added to the list of keywords. The most relevant key phrases from many research papers were chosen as initial keywords, such as “depression detection”, “depression diagnosis”, “MDD diagnosis”, and “mental illness disorders”. Other keywords, such as “EEG-based classification of depression”, “machine learning depression detection”, “EEG data depression detection”, and “deep learning depression diagnosis”, were chosen based on our understanding of the topic.

### 2.3. Data Sources

The academic databases utilized to find publications for the study are listed in Table 3.

### 2.4. Article Inclusion/Exclusion Criteria

According to the research objective, inclusion and exclusion criteria were devised to identify which papers should move on to the next review step. Articles that satisfied the criteria for inclusion were deemed pertinent to the study, while those that did not were eliminated. Table 4 provides examples of the inclusion and exclusion standards.

### 2.5. Article Selection

The article selection process involved three stages. First, 130 papers were considered after headings, abstracts, and terms were examined to find potentially pertinent publications. The second stage involved further analysis of the abstract, introduction, and conclusion, reducing the number of articles to 100. The remaining papers were carefully examined in the final and third stage, and their quality was evaluated based on how well they related to the research. Ultimately, 92 articles were selected for inclusion in this study.

## 3. Common Depression Diagnosis Pipeline

The process of diagnosing depression using EEG data involves a systematic pipeline designed to extract meaningful information from brain signals and accurately classify individuals as either depressed or non-depressed.

### 3.1. EEG Data Acquisition

The EEG signals, which are non-invasive and provide high temporal resolution, are critical for understanding brain activities and diagnosing conditions like depression. EEG signals are electrical potentials recorded from the scalp that reflect neuronal activity in the brain’s cortex. These signals are characterized by various features, such as amplitude, frequency, and power, which can be analyzed using linear and non-linear methods. EEG recordings typically use a standardized electrode placement system, such as the International 10–20 system, which ensures consistent and comprehensive coverage of different brain regions. For instance, electrodes are placed on the frontal, temporal, parietal, occipital, and central areas of the scalp to capture a broad range of brain activities [28].

The HydroCel Geodesic Sensor Net (HCGSN) is the most often utilized EEG gadget among the several used in different research projects to collect data [29]. The device’s 128 channels cover the entire brain. The second-most utilized EEG apparatus is the 64-channel neuroscan gadget [30]. In addition to this, Lanzhou University’s Ubiquitous Awareness and Intelligent Solution Lab (UAIS) has created a three-channel data-collection system. Two studies have employed this system with success.

### 3.2. Preprocessing

Since they might impair signal analysis and decision-making, it is crucial to deal with noise and artifacts in EEG data. Instead of being a result of cortical activity, such undesired noise might be created for various reasons, including experimental errors, ambient noise, and biological signal artifacts [31]. Several preprocessing techniques have been employed for studies focusing on MDD diagnoses, such as filtering and independent component analysis (ICA) multiple source modeling [32]. While other types of filters, such as band-pass, band-stop, low-pass, and high-pass filters, have also been widely employed with different cut-off frequencies, notch filters are frequently used to reduce interference from 50 Hz electrical lines [33]. Nonetheless, Kalman filters have not been thoroughly investigated in this area, and ICA and FastICA continue to be the most popular approaches.

### 3.3. Feature Extraction

The feature extraction process is of utmost importance as the models are not equipped to handle data with high dimensions, such as EEG signals, which may lead to overfitting [34]. Existing studies have used a variety of techniques to extract characteristics for a depression diagnosis [35,36,37,38]. Statistical, non-linear, spectral, and wavelet transform-based characteristics are the four categories into which our study subdivides these techniques. These features have been extracted using methods like fast Fourier transform (FFT), the autoregressive (AR) model, functional connectivity-based brain networks, non-linear analysis methods (like SODP, phase space trajectories, and others), statistical methods, and discrete wavelet transformation (DWT), wavelet packet decomposition (WPD), and EWT [39,40,41,42]. Of all the feature categories, non-linear analysis characteristics are employed the most frequently for both ML and DL approaches. The method in this category that has been most thoroughly studied is entropy-based computation. The EEG-based depression diagnosis utilizing the DL approach has not yet made use of geometrical features. Also, compared to the ML technique, statistical factors have not been considered as deeply for the DL strategy. Power and Hjorth parameters are the most often computed characteristics for the spectral category in ML and DL [43]. The methods in this field that use WT-based feature computations have received the least attention.

### 3.4. Feature Selection

Numerous techniques have been explored for feature selection and dimensionality reduction in EEG-based depression diagnosis [44]. The typically used approaches are principal component analysis (PCA) and genetic algorithms (GAs) [45]. However, studies utilizing the DL approach have not adequately studied feature selection and reduction methodologies. The DL-based technique is therefore thought to be capable of handling big datasets without the need for feature dimensionality reduction [46]. However, using automatic feature extraction in DL techniques has a disadvantage, as it can be challenging to identify specific features or biomarkers crucial for understanding the diagnosis process.

### 3.5. Classification

#### 3.5.1. Machine Learning Methods

Several ML methods, such as support vector machine (SVM)-based classifiers, including LS-SVM, SVM with various kernels, and CK-SVM, have been investigated for EEG-based depression detection [47]. There has also been research on discriminant analysis-based classifiers like the Gaussian mixture model (GMM) and BayesNet, ensemble designs like Bagging and RusBoost, probabilistic models like naive Bayes (NB) and logistic regression (LR), K-nearest neighbors (KNNs) and variations of it, tree-based algorithms like decision tree (DT), complex-tree, and J48, and others like linear discriminant analysis (LDA) and quadratic discriminant analysis (QDA) [48,49,50]. The SVM has been the most extensively researched traditional algorithm and has demonstrated excellent diagnostic performance [36,51,52]. Many people use KNN and modifications of it (weighted and enhanced), and they produce good results. Probabilistic models like LR and NB have received much attention for classification and have shown promise in several experiments. The most widely utilized ensemble model is random forest (RF), which has behaved well in a few trials. The most popular approach for classifying data using trees is called DT.

#### 3.5.2. Deep Learning Methods

**Figure 4:**
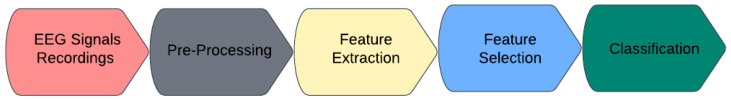
The general steps to detect depression using EEG signals.

The diagnosis of depression using EEG has been investigated using several deep learning algorithms, such as the convolutional neural network (CNN), HybridEEGNet, long short-term memory (LSTM), a combined CNN-LSTM algorithm, artificial neural network (ANN), multi-layer perception (MLP), radial basis function network (RBFN), MobileNet, ConvNet, DeprNet, Inception-v3, and ResNet-50 [5,46,53]. Three categories have been used to evaluate the performance of these architectures: deep neural networks, CNN-based architectures, and end-to-end architectures with different feature extraction techniques [54,55,56]. To diagnose depression using EEG, studies have primarily focused on LSTM and CNN-based designs. Additionally, end-to-end architectures have been used to directly process raw EEG signals, but their limitation is the lack of interpretability of the significant feature characteristics. Researchers have recently examined deep learning-based architectures and various feature extraction techniques, producing great classification results (Figure 4).

## 4. Depression Detection Literature Review

Several studies have explored the use of EEG signals for depression detection utilizing advancements in computational methods. For clarity and better analysis, these studies can be broadly categorized into two groups: those employing ML techniques and those utilizing DL approaches. This classification enables a focused discussion on the methodologies, strengths, and limitations of each group, highlighting the evolution of computational strategies in EEG-based depression detection.

### 4.1. Depression Detection Based on Conventional ML

Mumtaz et al. [57] suggested an ML model that utilized an EEG-derived synchronization likelihood (SL) dataset as input [58]. They employed LR, SVM, and NB classification models in their study. They connected study groups, such as MDD patients and healthy controls, and EEG properties. SVM classification accuracy was 98.0%, sensitivity was 99.9%, specificity was 95.0%, and f-measure was 97.0%; LR classification accuracy was 91.7%, sensitivity was 86.6%, specificity was 96.6%, and f-measure was 90.0%.

Sharma et al. [48] investigated the efficacy of different classifiers for EEG-based depression detection. The dataset comprised EEG signals from 30 subjects (15 normal and 15 depressed), with EEG recordings taken from both brain hemispheres at a sampling rate of 256 Hz. The EEG signals were preprocessed to remove artifacts and decomposed using a three-channel filter bank to extract significant features. These features were fed into various classifiers, including complex tree, LD, LR, bagged tree, KNN, and SVM. The study evaluated the performance of these classifiers using several metrics: classification accuracy (ACC), sensitivity (CSE), specificity (CSP), Matthews correlation coefficient (MCC), and area under the curve (AUC). Among the classifiers, the SVM with least squares optimization outperformed the others, achieving a CAC of 99.0% for left-hemisphere EEG signals and 99.54% for right-hemisphere EEG signals. They concluded that the right-hemisphere EEG signals yielded better classification performance, and the proposed SVM model demonstrated superior accuracy and robustness in distinguishing between normal and depressed subjects. The findings suggest that the proposed method can effectively classify depression using EEG signals with high accuracy.

Bachmann et al. [38] analyzed a 30-channel EEG signal and calculated the alpha power variability, spectral asymmetry index, and relative gamma power using linear and non-linear methods. Lempel–Ziv’s complexity was determined by calculating the Higuchi fractal dimension (HFD) and detrended fluctuation analysis (DFA). For classification, they employed LR analysis with leave-one-out cross-validation. The EEG streams were individually evaluated through separate measurements. The maximum accuracy for linear measures was 81.0%, while the maximum for non-linear measures was 77.0%. Combining two linear measures resulted in an accuracy of 88.0%, and combining two non-linear measures resulted in an accuracy of 85.0%. By combining three linear and three non-linear measures, the highest classification accuracy of 92.0% was achieved.

Cai et al. [49] gathered EEG signals from three locations on the forehead: frontal pole 2 (right side of the forehead), frontal pole zero (midline of the forehead), and frontal pole 1 (left side of the forehead) (Fp2, Fpz, and Fp1). Simplifying the test to only three channels may make diagnosing depression more accessible. The EEG signals from these areas were analyzed for their linear and non-linear properties in order to differentiate or recognize patients who are experiencing depression from those who are not. They evaluated non-linear properties like correlation dimension, C0 complexity, entropy types like Shannon, power spectrum, and Kolmogorov, and linear aspects like peak, variance, inclination, kurtosis, and the Hjorth parameter. They employed four techniques: gain ratio attribute evaluation, wrapper subset evaluation, correlation attribute evaluation, and principal components. They used five classification algorithms: LR, SVM, KNN, DT, and RF. They identified the most significant features using these algorithms. They delivered the best results among the four feature selection approaches for all classifiers, obtaining the best classification accuracy of 76.4%.

Mahato and Paul [42] explored various EEG-based methods for depression classification, utilizing both linear and non-linear features. The EEG data were processed with a sampling rate of 250 Hz and analyzed through different classifiers, including the multi-layer perceptron neural network (MLPNN), RBFN, LDA, and QDA. Key indicators such as accuracy, sensitivity, and specificity were employed to evaluate the models. Linear features like band power and interhemispheric asymmetry were initially tested, with alpha power and alpha asymmetry showing the highest classification accuracies of 91.6% and 73.3%, respectively. Non-linear features, particularly Renyi’s and wavelet entropies (RWE and WE), also demonstrated high classification accuracies of up to 90.0%. The combination of linear (alpha power) and non-linear features (RWE) achieved the highest classification accuracy of 93.33% in several classifiers. This study’s significant contribution demonstrates the effectiveness of combining linear and non-linear EEG features to improve depression classification accuracy.

Mahato and Paul [59] used features such as Alpha1, Alpha2, and Theta asymmetry, as well as Alpha, Alpha1, Alpha2, and Beta power. Multi-cluster feature selection (MCFS) was employed to choose features when a combination of characteristics was utilized. The classifiers employed were SVM, LR, NB, and DT. In all the classifiers tested, alpha2 demonstrated superior classification accuracy compared to alpha1 and alpha power. Alpha2 and theta asymmetries together produced the best accuracy for classification in the SVM, coming in at 88.3%.

Saeedi et al. [46] extracted five frequency bands as linear characteristics, including theta, delta, alpha, gamma, and beta. Additionally, they employed WPD to divide signals into particular frequency bands. The wavelet packet coefficients were used as non-linear features in two entropy applications: sample entropy and approximate entropy. A genetic algorithm was used to choose the pertinent traits. Three methods for machine learning were utilized for classification: SVM, MLP, and their enhanced KNN (E-KNN) approach. These algorithms used the GA to reduce feature space distances and generate an index of feature importance. Gamma oscillations’ frequency-based properties allowed for a maximum accuracy of 91.3%. With a 94.2% accuracy rate, non-linear features fared better than frequency-based features.

Cai et al. [53] used a three-electrode EEG setup at the Fp1, Fp2, and Fpz electrode locations. EEG data were obtained from participants during rest and sound exposure. After reducing noise, they recovered 270 linear and non-linear characteristics with the discrete impulse response filter, which combines the Kalman derivation method, DWT, and an adaptive predictor filter. The mRMR feature selection approach was used to minimize the dimensionality of the feature space. Depressed subjects and control participants were separated using four classification techniques: SVM, K-NN, ANN, and classification trees. Ten-fold cross-validation was used to assess the performance of the classifiers, with K-NN achieving the greatest accuracy of 79.2%.

Akbari et al. [60] developed a technique for diagnosing depression that makes use of geometric aspects that may be derived from the SODP’s form. With the aid of BPSO, an appropriate feature selection was made. To identify between normal and depressed signals, SVM and K-NN classifiers were trained using the chosen characteristics. Bipolar EEG readings from 22 healthy subjects and 22 patients with MDD were used to evaluate the approach. Using a ten-fold cross-validation technique and the city block distance metric, the KNN classifier obtained an accuracy (ACC) of 98.7%.

Akbari et al. [60] suggested a unique method for identifying depression utilizing geometrical features and the RPS of the EEG. They used some optimization algorithms, including the GA, ant colony, grey wolf, and particle swarm optimization (ACO, GWO, and PSO), with the GA scoring the best at 58.8%. The chosen features were then input into two classifiers: the KNN classifier, which uses Euclidean and city block distances, as well as the SVM classifier, which uses a radial basis function (RBF) kernel. Using the PSO’s chosen characteristics in a 10-fold cross-validation, the suggested framework achieved a high ACC of 99.3% and an MCC of 98.0%.

Peng et al. [61] aimed to see if depressed individuals’ resting-state functional connectivity patterns had changed in any way and if these modifications could determine the differences between them and healthy people. To generate functional connection matrices, the researchers employed the phase lag index, and to find features with strong discriminative strength, they adjusted the KRCC. To categorize 27 depressed subjects and 28 healthy control subjects who were matched demographically, they used various classifiers. The effectiveness of the classifiers was assessed using permutation tests. With leave-one-out cross-validation and a 98% for AUC, a binary linear SVM produced the best results, correctly classifying over 92.0% of subjects throughout the entire frequency band.

Liu et al. [37] provided a viable method for determining the presence of serious MDD and provided insights into the neural circuits involved in music perception in MDD patients. Compared to healthy individuals, the study found that patients with MDD showed decreased connectivity in the delta frequency range but increased connectivity in the beta frequency range while listening to music. Additionally, healthy individuals exhibited left-hemisphere dominance, which was not observed in MDD patients. With accuracy, sensitivity, and specificity of 89.7%, 89.4%, and 89.9%, the SVM performed the best in the beta frequency band.

Zhu et al. [18] proposed the CBEM, a revolutionary ensemble technique, to enhance the precision of identifying depression. The proposed approach involves static as well as a dynamic CBEM. Using this approach, the subjects’ labels are decided by a majority vote after the EEG or Ems dataset has been partitioned into subgroups based on the research context. Using two datasets, eye-tracking data and resting-state EEG data, respectively, made up of 36 and 34 people each, the efficacy of the suggested approach was validated. With accuracy rates of 82.55% and 92.65% for these two datasets, the CBEM outperformed more established categorization techniques.

Aydemir et al. [62] proposed an automated system for detecting MDD using EEG signals. The process includes various stages, such as creating features by utilizing a melamine pattern and DWT, choosing features through neighborhood component analysis, and categorizing using SVM and k-NN classifiers. What distinguishes this system from others is the utilization of a melamine structure. This design’s molecular structure produces 1536 characteristics, and statistical attributes are obtained from DWT coefficients. When utilizing the A2A1 EEG channel, the system obtained the highest classification accuracy of 99.1% and 99.0% by using a weighted k-NN and quadratic SVM, respectively.

Movahed et al. [50] suggested an ML approach for assessing MDD that utilizes a range of features derived from EEG data, including functional connectivity, statistical, wavelet, spectral, and non-linear analysis methods. They also performed feature selection using the SBFS algorithm. The method was tested on a publicly available EEG dataset, using 10-fold cross-validation to obtain performance metrics such as the F1 score, specificity, accuracy, sensitivity, and false discovery rate. Results show that the proposed method achieved an AC of 99.0%, a sensitivity of 98.4%, a specificity of 99.6%, an F1 score of 98.9%, and a false discovery rate of 0.4% when using the SVM with an RBF kernel classifier.

Kaur et al. [63] aimed to reduce the noise in EEG signals related to depression. To evaluate their efficacy, the scientists contrasted two denoising techniques that rely on DWT and WPT with VMD. Detrended fluctuation analysis was used to choose the best denoising modes. The signal was initially divided into several components using VMD. Then, instead of completely rejecting the artifactual components using DFA as the mode selection criterion, their noise was reduced using DWT and WPT. Performance metrics, including (peak) signal-to-noise ratio (SNR, PSNR), and mean squared error (MSE), were used in simulations using genuine depression databases as well as intentionally contaminated databases to show how effective the study was.

Duan et al. [36] processed the EEG signals and divided them into segments. Then, the structural characteristic matrix was modified to create mixed features by adding and removing the feature matrix that included connectivity features and frequency bands. The three classifiers were then utilized to determine the proper features for classification, and it was discovered that mixed features produced the best classification outcomes. According to the study conducted at Beijing Anding Hospital by Capital Medical University, the method that was suggested achieved an accuracy rate of 94.1%, a sensitivity rate of 95.7%, a specificity rate of 93.5%, and an F1 score of 95.6% when using the data.

Vcukic et al. [35] employed HFD and SampEn as features in seven machine learning algorithms—MLP, LR, DT, SVM with linear and polynomial kernels, RF, and NB classifiers—to distinguish between EEG data from healthy and depressed people. The study affirmed previous discoveries that both non-linear metrics could differentiate EEG signals obtained from healthy individuals and patients. The findings indicate that achieving a satisfactory classification outcome is possible even when using a limited number of principal components. The classifiers achieved an average accuracy of 90.2% to 97.5%.

Jiang et al. [51] suggested an approach that considers geographical information while analyzing EEG signals to detect depression. During the study, 30 individuals took part in a task that required them to identify emotive facial expressions in a group of people. Out of the 30 participants, 16 were diagnosed with depression, while the other 14 were considered to be in good health. For feature extraction and selection, they employed differential entropy and the genetic method; for classification, they used the SVM. Before feature extraction, they included a task-related common spatial pattern to reduce the spatial differences. When TCSP was used, the authors discovered that classification accuracy was higher than when it was not. In particular, their cross-validation accuracy achieved a rate of 84.0% for positive stimuli and 85.0% for negative stimuli when leaving out one subject. This was statistically greater than the 81.7%.

Akbari et al. [52] introduced a technique to distinguish between normal and depressed EEG data based on centered correntropy and EWT. The EWT was utilized to separate the EEG signals into rhythms, and the CC of these rhythms was calculated as the distinctive feature and sent to classifiers like K-NN and the SVM. EEG waves from 22 healthy participants and 22 depressed subjects were used to assess the proposed method. An SVM classifier was utilized to acquire the ACC, sensitivity, and specificity of 98.7%, 98.4%, and 99.0%, respectively, using a 10-fold cross-validation technique. According to these effective results, the proposed method may be a quick and reliable computer-aided diagnostic tool for identifying depression patients in clinical and hospital settings.

Tasci et al. [64] introduced a classification model that was both self organized and computationally efficient, explicitly designed for the detection of MDD with a high level of accuracy. The model was created manually and validated using a strategy based on reference subjects. The researchers utilized the MODMA public dataset, which includes EEG signals from 128 channels. The dataset consisted of recordings from 24 individuals with MDD and 29 healthy controls. A pattern called twin Pascal’s triangles lattice pattern (TPTLP) consisting of 25 values was employed to capture textural details from the original EEG signal and its sub-bands. During each run, we extracted forty statistical characteristics at the same time. They used neighborhood component analysis to select the essential features and obtained 128 prediction vectors for each channel using the k-NN classifier. The created model showed the most accurate results overall and for individual channels. When tested using the leave-one-subject-out cross-validation method, the system achieved an accuracy of 76.0% for Channel 1 and 83.9% when the results from the top 13 channels were combined. When using a 10-fold cross-validation technique, the accuracy was 100.0%.

Liu et al. [65] investigated using EEG data to improve the diagnosis of MDD through advanced machine learning techniques. The study employed a novel W-GCN-GRU model, which integrates graph convolutional networks (GCNs) and gated recurrent units (GRUs), to classify EEG recordings into depressive and non-depressive states, achieving high classification accuracy with a test set accuracy of 94.5%. Logistic regression was utilized to identify significant EEG predictors, such as beta2 power in the prefrontal cortex with eyes open, enhancing feature selection and model interpretability. This combined approach highlights the potential of EEG-based biomarkers and sophisticated neural network models in providing accurate and objective tools for the diagnosis and personalized treatment of depression.

Noda et al. [66] introduced the efficacy of using TMS-EEG data to diagnose MDD. EEG data were analyzed for the frontal region’s power spectrum, phase synchronization, and phase-amplitude coupling in various frequency bands (α, β, γ, θ). Indicators included average power values, weighted phase lag index (wPLI) for phase synchronization, and modulation indices for phase-amplitude coupling. Nine machine learning models were employed, including LR, SVM, RF, and lightGBM, to classify the data from 60 MDD patients and 60 healthy controls. The best performance was achieved using LDA, yielding a mean AUC of 92.2%, indicating high accuracy. This research highlights the potential of integrating TMS-EEG data with advanced ML techniques to develop reliable diagnostic tools for depression, significantly contributing to the field by offering a non-invasive and objective method for identifying depressive states.

Khan et al. [67] developed an EEG-based framework to detect MDD. They utilized the MODMA dataset, which includes EEG data from 55 participants recorded using three electrodes in a resting-state condition. The researchers extracted twelve temporal domain features from the EEG data using non-overlapping 10-s windows. These features were subjected to a novel feature selection mechanism to identify the most discriminative attributes. Three classification algorithms were employed, namely, best-first (BF)-Tree, KNN, and AdaBoost. The BF-Tree classifier achieved the highest accuracy of 96.36%, surpassing existing state-of-the-art methods in terms of the number of electrodes used, feature vector length, and overall classification accuracy. The study highlighted the potential of their proposed framework to be used in psychiatric settings, providing valuable support to psychiatrists for the diagnosis of depression (Table 5).

**Table 5:** A summary of studies based on machine learning.

Study	Features	Method	Dataset	Accuracy
Mumtaz et al. [57]	EEG-derived synchronization likelihood (SL)	SVM, LR, and NB	34 depressed 30 normal	98.0%, 91.0%, and 93.0%
Sharma et al. [48]	WSBs of EEG signal	LS-SVM	15 depressed 15 normal	99.54%
Bachmann et al. [38]	Spectral asymmetry index, alpha and gamma power	Logistic regression	13 depressed 13 normal	92.0%
Cai et al. [49]	Peak, variance, inclination, kurtosis, entropy	KNN, SVM	152 depressed 113 normal	76.4%
Peng et al. [61]	Delta, theta, alpha, and beta with high discriminative power	SVM	13 depressed 13 normal	92%
Liu et al. [37]	Delta and beta	SVM	19 depressed 20 normal	89.7%
Zhu et al. [18]	Delta, theta, alpha, and beta	CBEM	17 depressed 17 normal	92.6%
Saeedi et al. [46]	Delta, theta, alpha, beta, gamma, entropy	E-KNN	34 depressed 30 normal	98.4%
Mahato and Paul [59]	Alpha, alpha1, alpha2, beta, delta, theta	SVM, LR	34 depressed 30 normal	88.3%
Duan et al. [36]	Structural and connectivity features	KNN, SVM	16 depressed 16 normal	83.1%, and 88.2%
Vcukic et al. [35]	HFD, sample entropy	NB, LR, MLP, SVM, DT, and RF	23 depressed 20 normal	90.2% to 97.5%
Aydemir et al. [62]	Statistical features from DWT coefficients	Weighted kNN and quadratic SVM	34 depressed 30 normal	99.1%, and 99.0%
Movahed et al. [50]	Statistical, spectral, and non-linear features	RBFSVM	34 depressed 30 normal	99%
Jiang et al. [51]	Delta, theta, alpha, and beta	SVM	16 depressed 14 normal	84%, 85.7% for ±stimuli
Akbari et al. [60]	EEG-reconstructed phase space, geometrical features	KNN, SVM	22 depressed 22 normal	99.3%
Tasci et al. [64]	Statistical features	KNN	24 depressed 29 normal	76.8%
Liu et al. [65]	Beta1, alpha, and theta power bands	W-GCN-GRU	86 depressed 83 normal	94.5%
Noda et al. [66]	Beta power, gamma phase synchronization, alpha and theta phase synchronization	LDA, LR, SVM, KNN, RF, ET, NB, LG, DT	60 depressed 60 normal	LDA: 92.2% (AUC), SVM: 90.1%, KNN: 88.3%
Khan et al. [67]	Temporal domain features (mean, variance, skewness, kurtosis)	KNN AdaBoost BF-Tree	MODMA: (29 healthy, 26 depressed)	KNN: 94.7%, AdaBoost: 79.0%, BF-Tree: 97.0%

### 4.2. Depression Detection Based on DL

Mahato and Paul [42] used different features to classify a dataset of 34 depressed participants and 30 healthy controls. Both linear and non-linear characteristics, such as RWE and a mix of the two, were included in the features. Examples of linear characteristics include band power and interhemispheric asymmetry. Mumtaz et al. contributed to the dataset that was used in the investigation. The study used two types of classifiers: the MLP neural network (MLPNN) and radial basis function network (RBFN). When using linear characteristics, the alpha power of the MLPNN classifier achieved the highest classification accuracy of 91.67%. When using non-linear features, both RWE and a combination of linear and non-linear features achieved 90.0% classification accuracy with the RBFN. Combining linear and non-linear properties with MLPNN and RBFN classifiers led to the most significant classification accuracy of 93.3%.

Saeedi et al. [46] introduced a technique to differentiate between depressed patients and healthy controls using short-term EEG signals. To achieve this, linear characteristics of the signals in five frequency bands (delta, alpha, beta, theta, and gamma) were defined. The signals were divided into several frequency bands using wavelet packet decomposition, and these frequency bands were then subjected to two entropy measures (sample entropy and approximation entropy) to obtain non-linear features. The data were categorized using MLP, and relevant features were chosen using a genetic algorithm. Gamma oscillations were used as frequency-based characteristics to reach the greatest accuracy of 91.3%. With a result of 94.2% accuracy, non-linear features surpassed frequency-based features.

Acharya et al. [68] introduced a computer model for diagnosing depression that uses the convolutional neural network (CNN) or deep neural network approach. By automatically learning to distinguish between EEG signals recorded from sad and normal participants, this unique technique does away with the need for manually choosing a set of criteria for categorization. EEG signals from 15 healthy individuals and 15 patients who were depressed were used to assess the model. The program generated EEG signals from both the left and right hemispheres with an accuracy of 93.5% and 96.0%, respectively. According to the study’s findings, depression is more differentiated in right-hemisphere EEG signals than in left-hemisphere data.

Sharma et al. [5] introduced a hybrid neural network approach for diagnosing depression called the Depression Hybrid Neural Network. LSTM architectures were employed for sequence learning, while CNN architectures were used for temporal learning and windowing. To evaluate the model, EEG data from 21 symptomatic depressed patients who were drug-free and 24 healthy patients who underwent a neuroscan were used. The windowing technique was used to reduce computational complexity and processing time. The mean absolute error of the model was 0.2040, and its accuracy was 99.1%. The study’s conclusions showed that the hybrid CNN-LSTM model had been established and could be used to accurately identify depression in EEG signals.

Ay et al. [69] proposed a novel deep hybrid model using EEG waves for identifying depression that combined CNN and LSTM architectures. The deep model was designed to learn the temporal characteristics of the signals through CNN layers and sequence learning through LSTM layers. EEG signals from both the left and right hemispheres were used in this study. The classification accuracy for the right hemisphere was 99.12%, while that for the left hemisphere was 97.66%. In light of the results, it can be said that the CNN-LSTM model can quickly and correctly identify depression in EEG signals.

Thoduparambil et al. [70] developed a deep model to identify depression by combining a CNN and LSTM. While LSTM was used to learn the signal sequence, the CNN was utilized to learn the local properties of the EEG signals. Filters convolved the incoming signal and created feature maps in the CNN layer. After learning various patterns, the LSTM layer received all of the chosen features and used fully linked layers to classify the signal. The LSTM layer included memory cells that allowed it to recall essential features for an extended period of time. This model had accuracy rates of 99.07% for right hemisphere signals and 98.84% for left hemisphere signals.

Seal et al. [71] demonstrated the DeprNet, a DL-based CNN model, can be used to categorize EEG data from depressed and healthy participants. The level of depression was measured using the Patient Health Questionnaire 9 score. The model’s effectiveness was assessed under two separate hypotheses: record-wise split and subject-wise split. DeprNet achieved a high level of accuracy, equal to 99.3%, and an AUC value of 99.9% when the data were divided on a per-record basis. In contrast, if the data were split by subject, the accuracy decreased to 91.4%, and the AUC decreased to 95.6%.

Mumtaz and Qayyum [72] suggested a pair of DL models to differentiate between depressed participants and healthy subjects based on EEG recordings. Specifically, they developed a 1 Dimension-CNN (1DCNN) and a hybrid model consisting of a 1DCNN and LSTM. These models were able to learn patterns in the EEG signals and identify significant differences between the two groups. The CNN model achieved a high classification accuracy of 98.3%, a precision of 99.7%, a recall of 98.3%, and an f-score of 97.6%. The LSTM-based 1DCNN model also performed well, with a classification accuracy of 95.9%, a precision of 99.2%, a recall of 93.6%, and an f-score of 95.1%. The authors concluded that their DL framework has the potential to automatically diagnose depression.

Wan et al. [56] revealed HybridEEGNet, a CNN with two parallel lines that can discriminate between control participants, patients with MDD who are on medication, and those who are not. They tested and trained the model using a ten-fold cross-validation technique. In the three-category categorization, the HybridEEGNet attained a sensitivity of 68.7%, a specificity of 84.4%, and an accuracy of 79.0%. According to the EEG feature analysis, variations in the alpha rhythm’s spatial distributions and amplitude ranges (about 10 Hz) between the three groups may be the key indicators for diagnosing depression.

Saeedi et al. [73] suggested a DL model to distinguish between healthy people and MDD patients based on EEG. They started by carefully studying the relationships between the channels in the EEG data using two techniques: generalized partial directed coherence (GPDC) and directed transfer function (DTF). Each individual’s EEG data were individually portrayed using 16 connection techniques (GPDC and DTF) in eight frequency bands. The produced EEG signal pictures were then subjected to one of five deep-learning models. In the first two techniques, CNNs in one and two dimensions, respectively, were used. The third method used the LSTM model to handle lengthy short-term memory, which integrated the CNN and LSTM models. The 1DCNN-LSTM and 2DCNN-LSTM were the fourth and fifth approaches, respectively. The study found that the 1DCNN-LSTM model, which achieved an accuracy of 99.2% when applied to a generated image of effective connectivity, was the most effective strategy. The architecture that captured the spatial and temporal relationships in brain connectivity was to blame. This technique can assist clinicians in swiftly diagnosing patients with MDD, enabling prompt treatment and diagnosis.

Uyulan et al. [74] built a diagnosis model for MDD using modern computational neuroscience methods, a deep CNN, and EEG. To evaluate EEG recordings and differentiate between those with MDD and healthy controls, MobileNet, ResNet-50, and Inception-v3 are three different deep CNN architectures that the researchers employed. Several DL architectures were employed to highlight the discrimination capabilities by contrasting classification accuracy. Classification accuracy for models built utilizing location data was 89.3% and 92.6%, respectively. With an AUC value of 0.9 and a prediction accuracy of 90.2% for the ResNet-50 architecture, the delta frequency band fared better than other frequency bands.

Loh et al. [34] created a DL model to analyze EEG data to identify MDD. Using the EEG data of 34 MDD-depressed participants and 30 healthy people, they created spectrogram images using STFT. Using these spectrogram images, the CNN technique was then used to automatically distinguish between MDD-depressed individuals and healthy people. Using hold-out validation, 99.5%, 99.4%, 99.7%, 99.4%, and 99.5%, respectively, were the model’s high classification accuracy, precision, sensitivity, and specificity values. To confirm the model’s generalizability, the researchers pointed out that it has to be validated on a bigger and more varied MDD database.

Li et al. [75] suggested a CNN-based computer-aided detection system. They emphasized the significance of developing them on a local database to ensure that the CAD system and ConvNet architecture can be used in clinical practice. They used transfer learning to build the ConvNet architecture. Also, they looked into the importance of other EEG characteristics, including spectral, spatial, and temporal information, and discovered that the former two considerably increased accuracy. The training and test sets were divided depending on individuals using 24-fold cross-validation. The suggested strategy produced an 85.6% accuracy for differentiating between moderate depression and normal controls.

Li et al. [40] introduced a brand categorization approach for spotting mild depression early on. Due to the CNN’s robust two-dimensional data processing capabilities, they independently utilized it to create the functional connectivity matrices from each of the five EEG bands (delta, theta, alpha, beta, and gamma). Additionally, they used the CNN to separate moderate depression-related and normal EEG data by combining the functional connectivity matrices from the three best-performing EEG bands. Recent advancements in the ability of deep recurrent CNNs to categorize mental strain served as inspiration for this. A classification accuracy of 80.7% was achieved by the proposed classification model for recognizing moderate depression.

Khan et al. [4] suggested a method for automatically detecting MDD using a 2D-CNN network and a newly developed biomarker. The biomarker was created by analyzing wavelet coherence (WCOH) among some areas of the brain’s DMN through EEG signals. For the network to be trained and assessed for its performance, 30 MDD participants and 30 control subjects were randomly divided into training and testing sets for the study. By evaluating the network’s performance solely on the training data, 10-fold cross-validation was employed to prevent learning bias. The subjects were tested using two classification techniques: sample-based and subject-based. The results showed that the sample-based approach achieved an accuracy of 98.1%, a sensitivity of 98.0%, and a specificity of 98.2%. The second option provides a perfect score of 100.0% for accuracy, sensitivity, and specificity.

Tigga and Garg [76] suggested a model called AttGRUT to effectively detect disturbances in the EEG waves of depressed patients. The 60-channel signal from the EEG data was first processed to extract statistical, spectral, and wavelet characteristics. The key characteristics were then chosen by utilizing three feature selection techniques: Shapley additive explanations, recursive feature removal, and the Boruta algorithm. The suggested approach performed better than four other time-series models, including two standards and two mixed models: LSTM, GRU, CNN-LSTM, and CNN-GRU. The suggested model reached an accuracy level of 98.6%. By selecting the most important features, the performance of all the time-series models improved significantly.

Ying et al. [77] proposed a novel deep learning model named Enhanced Depressive Tendency (EDT) for depression recognition using EEG data. The EDT model was designed to effectively capture depression-specific information in the frequency, spatial, and temporal domains of EEG signals. It consisted of an information extraction module and an attention module, which together extracted and integrated multi-domain features. The study utilized EEG data from two sources: an 18-channel dry electrode cap and a 128-channel HydroCel geodesic sensor network. The model was evaluated using various metrics and demonstrated superior performance over baseline models and its variants. Notably, the EDT model achieved a high classification accuracy of 94.0%, underscoring its effectiveness in identifying depressive states. This research significantly advanced EEG-based depression recognition by integrating multi-domain features with advanced attention mechanisms, highlighting its potential for practical applications in mental health diagnostics.

Zhang et al. [78] proposed a novel model called the Sub-domain Splitting and Pooling Graph Convolutional Network (SSP-GCN) for EEG-based depression recognition. Utilizing EEG data from the MODMA and PRED + CT datasets, which included signals sampled at 250 Hz and 500 Hz, respectively, with 128 and 66 channels, the researchers focused on several indicators: accuracy, recall, precision, F1 score, and the Polygon Area Metric (PAM). The study evaluated various classifiers, including the SVM, CNN, EEGNet, GCN, GCN with domain generalization (DG), and the proposed SSP-GCN model. The SSP-GCN model demonstrated superior performance, achieving the highest classification accuracy of 92.8% on the MODMA dataset and 83.1% on the PRED + CT dataset. This model’s key innovation lies in its effective utilization of topological information between EEG channels and its incorporation of domain generalization techniques to address cross-subject variability, resulting in significant improvements in classification accuracy. The introduction of the SSP-GCN model represents a substantial advancement in the field, highlighting its potential for enhanced diagnostic accuracy in EEG-based depression detection (Table 6).

**Table 6:** The summary of the discussed studies based on deep learning.

Study	Deep Learning Network	Dataset	Accuracy
Acharya et al. [68]	CNN	15 depressed 15 normal	left: 93.5% right: 95.9%
Mahato and Paul [42]	MLPNN	34 depressed 30 normal	93.3%
Mumtaz and Qayyum [72]	CNN 1DCNN-LSTM	33 depressed 30 normal	98.3% 95.9%
Ay et al. [69]	CNN-LSTM	15 depressed 15 normal	left: 97.6% right: 99.1%
Li et al. [75]	ConvNet	24 depressed 27 normal	85.6%
Thoduparambil et al. [70]	CNN-LSTM	46 depressed 75 normal	right: 99.0% left: 98.8%
Wan et al. [56]	HybridEEGNe	11 depressed 12 normal 12 unmedicated	79.0%
Li et al. [40]	CNN	24 depressed 27 normal	8.7%
Ay et al. [5]	Hybrid CNN-LSTM	21 depressed 24 normal	99.1%
Sharma et al. [71]	DeprNet	18 depressed 15 normal	subjectwise split data: 91.4% recordwise split data: 99.3%
Saeedi et al. [73]	1DCNN-LSTM	34 depressed 30 normal	99.2%
Uyulan et al. [74]	ResNet-50 MobileNet Inception-v3	46 depressed 46 normal	left: 75.55%, 89.33%, 67.88% right: 87.6%, 92.6%, 77.6%
Loh et al. [34]	CNN with spectrogram image	34 depressed 30 normal	99.5%
Khan et al. [4]	2D-CNN	30 depressed 30 normal	sample-based: 98.1% subject-based: 100.0%
Tigga and Garg [76]	AttGRUT	-	98.6%
Ying et al. [77]	EDT	-	94.0%

## 5. Discussion and Comparison

In this discussion, we provide a comprehensive analysis of the studies in the Systematic Literature Review (SLR), focusing on the methodological approaches and findings that shape the current landscape of EEG-based depression detection. The previous sections outlined the procedure for study selection and detailed the five-step framework used for analyzing each research work. Here, we present a statistical overview of the reviewed articles, highlighting key trends, strengths, and limitations across studies. The analytical questions introduced in Section 2 are addressed, offering evidence-based answers that synthesize the findings from the literature. This discussion aims to critically assess the overall body of evidence, identify gaps and inconsistencies, and suggest future directions for research based on the comprehensive analysis of the available data.

- **AQ1:** What ML and DL models are most effective in classifying EEG signals for depression diagnosis?
  From the analysis, ML approaches such as the SVM consistently show high performance, particularly when paired with advanced features. For example, Mumtaz et al. [57] achieved an accuracy of 98.7% using EEG-derived synchronization likelihood features with the SVM, while Peng et al. [61] reached 97% accuracy using delta, alpha, and beta power features with KNN/SVM. Other models like KNN and decision trees demonstrate moderate accuracy, typically around 70–85%, such as the study by Tasci et al. [64], which reported 74.67% accuracy.
  For DL approaches, CNN-based models dominate in performance. Thodupunuri et al. [70] achieved an accuracy of 98.8% with a CNN-1LSTM model, and Khan et al.’s [4] 2D-CNN achieved 98.1% accuracy using a spectrogram image of the EEG data. Hybrid models like the CNN-LSTM (e.g., Ay et al. [69]) further highlight the advantage of combining spatial and temporal features, reaching 93.4% accuracy. However, models like EDT (Ying et al. [77]) show variability, achieving 94% but requiring large datasets to optimize. While ML models are effective with strong feature engineering, DL models show superior performance, especially when utilizing raw data and large datasets.
- **AQ2:** How do various EEG preprocessing methods impact the performance of ML and DL models in depression detection?
  Preprocessing techniques heavily influence the performance of both ML and DL models. Studies incorporating advanced EEG preprocessing, such as power spectral density analysis (e.g., Peng et al. [61], 97%) and artifact removal (e.g., Mumtaz et al. [57], 98.7%), demonstrate significantly higher accuracies compared to those using minimal preprocessing. For DL models, preprocessing methods such as converting EEG signals into spectrogram images (e.g., Khan et al. [4], 98.1%) or segmenting signals into frequency bands (e.g., Sharma et al.’s [71] DeprNet, 91.4%) enhance performance. While DL models can handle raw data, preprocessing reduces noise and enhances interpretability, leading to more robust results. Minimal preprocessing, while feasible for DL models, generally results in lower accuracy or longer training times.
- **AQ3:** What are the primary EEG signal features and biomarkers utilized for depression detection?
  The analysis reveals that EEG biomarkers like alpha, beta, and theta band power are the most commonly used features for depression detection. For instance, Peng et al. [61] utilized delta, alpha, and beta power features to achieve 97% accuracy with the SVM/KNN. Similarly, Saeedi et al. [46] leveraged beta and theta alpha feature for an accuracy of 89.8% using KNN.
  For DL models, raw EEG signals and spectrograms are preferred to allow models to extract features automatically. Models like the CNN and 1DCNN-LSTM use raw EEG or time-series data, as seen in Thodupunuri et al. [70] that achieved an average accuracy of 98.8%. This approach enables the models to learn spatial and temporal patterns, bypassing manual feature engineering.
- **AQ4:** What challenges and research gaps exist in using EEG for depression detection and diagnosis?
  Despite significant advancements in EEG-based depression diagnosis, several challenges persist, and key research gaps remain that require further investigation to advance the field.
**Challenges**
  One significant challenge is analyzing and interpreting EEG data in complex cognitive tasks like music perception. Understanding how mental disorders impact brain oscillations in more naturalistic settings, such as during everyday activities, remains a difficult yet critical area for research. Additionally, the complexity of EEG signals often requires advanced techniques like EEG source localization to explore brain alterations in individuals with major depression [57].
  Another major challenge lies in identifying consistent and reliable biomarkers for distinguishing between depressive episodes and remission. While non-linear EEG biomarkers capturing physiological complexity are being explored, their utility in practical diagnostic systems is still under development [79].
**Research Gaps**
  Several critical research is identified, as follows:
  - **Naturalistic EEG Studies:** There is a lack of research investigating EEG patterns in real-world, naturalistic scenarios, such as during everyday activities or social interactions, which could provide more meaningful insights into brain dynamics in depression.
  – **Biomarkers for Transition States:** While EEG biomarkers for depression are studied, there is insufficient research on biomarkers that can reliably differentiate between episodes of major depressive disorder (MDD) and remission [79].
  – **Distinguishing Bipolar vs. Unipolar Depression**: Limited studies focus on using EEG biomarkers or machine learning models to accurately distinguish bipolar depression from unipolar depression. This is a critical gap, given the differences in treatment approaches for these conditions [80].
  – **Dataset Size and Diversity:** A significant research gap exists in the availability of large-scale, diverse, and standardized EEG datasets for depression diagnosis. Current datasets are often too small or lack variability, limiting models’ generalizability and real-world applicability.
  – **Generalized ML/DL Models:** Most machine learning and deep learning models are designed for specific datasets and perform poorly across different populations or scenarios. Research is needed to develop more generalized models that are robust across varying EEG data and depression subtypes.

## 6. Datasets

The scientists obtained the data either from publicly available online repositories or by collecting them from local sources. Table 7 summarizes the studies that used different public and local EEG datasets to diagnose depression using two different approaches. The following is a description of some common datasets, which include the number of study subjects, the number of EEG channels, the data sampling rate (in Hz), the reference electrode, the study reference that used the relevant dataset, the source from which the data were collected (or a URL link for a publicly available dataset), the number of participants in the study, and the specific diagnostic approach used.

Some studies [34,42,46,57,59,62,73] used the dataset that was prepared by [81] which is accessible to the general public (https://figshare.com/articles/EEG_Data_New/4244171, accessed on 27 November 2024). In addition, 34 depressive patients (17 females) with ages ranging from 27 to 53 were identified, along with 30 normal individuals (9 females) with ages ranging from 22 to 53. With the eyes closed, EEG data were gathered for five minutes. Records were taken from 19 electro-cap electrodes placed on the scalp and classified using the 10–20 international standard electrode position classification method. Furthermore, sampling at 250 hertz was carried out. A notch filter was used to remove the 50 Hz power line noise. Between 0.5 and 70 Hz, band-pass filters were applied to all EEG signals.

Other studies, such as [68,69], employed the dataset supplied by the psychiatry department of the medical college in Calicut, Kerala, India. The data were submitted by 15 subjects (20–50 years old, 15 healthy, and 15 depressed). With eyes open and closed, bipolar EEG signals were recorded for 5 min from the left (FP1-T3 channel pairs) and right (FP2-T4 channel pairs) halves of each subject’s brain (resting state). The EEG signals were recorded at 256 Hz using a 50 Hz notch filter to eliminate interference from power lines.

On the other hand, Thoduparambil et al. [70] used the dataset provided by PRED+CT [82]. The dataset comprises EEG data acquired using 64Ag/A electrodes and Synamps2 system voltage from the scalp of the brain (500 Hz sampling frequency, band-pass filter 0.5–100, conductivity less than 10 k).

**Table 7:** The common public datasets for EEG-based depression diagnoses.

Dataset Name	The Reference	Patient Group	EEG Task	Patient Number	EEG System	Electrodes Number
Healthy Brain Network	[83]	Depressive	Resting State	10,000	128 EEG channel	128
EMBARC	[84]	Depressive	Resting State	675	—	16
Depresjon	[85]	Depressive	Motor Activity	55 23D+32H	—	—
Transdiagnostic Cohorts	[86]	Depressive	—	287	—	—
MIPDB	[87]	Depressive	Resting State	126	—	109
PREDICT	[82]	Depressive High BDI	Reinforcement	46	Neuroscan	64

### 6.1. Healthy Brain Network (HBN)

The HBN is a public data biobank established by the Child Mind Institute [83]. HBN’s primary purpose is to provide a dataset that reflects the vast range of variance and impairment as psychopathology progresses. The HBN uses multi-modal EEG and magnetic resonance imaging (MRI) brain imaging to collect data on depressive disorders and behavioral, intellectual, eye-tracking, and phenotypic information. In the HBN, mental health and learning issues are assessed clinically. The HBN methods include behavioral and physical exams, family structure, anxiety, cognition, trauma, and linguistic problems. The data were collected from 10,000 New York citizens between the ages of 5 and 21.

### 6.2. EMBARC

The EMBARC dataset is a free accessible dataset created by the NIMH [84]. Using a machine learning algorithm and a resting-state EEG, it finds neurological signs of an antidepressant effect. P1, P2, P3, PO3, P4, P5, POz, P6, P7, P8, PO4, PO7, PO8, Oz, O1, and O2 were the 16 posterior electrodes.

### 6.3. Depresjon

Depresjon [85] utilizes recordings of motor activity from 32 normal subjects and 23 patients with unipolar and bipolar depression, as well as a publicly available dataset of motor activity.

### 6.4. Transdiagnostic Cohorts

Transdiagnostic cohorts [86] is a dataset that evaluates the effectiveness of short, transdiagnostic psychological group therapy for anxiety and depressed patients is publicly available. It includes 287 anxiety disorders and depression patients from primary care. These individuals received TCBGT for around five weeks. ANOVA tests with mixed specifications were employed for statistical analysis and to confirm the therapeutic effects.

### 6.5. MIPDB

It focuses on dimensional and multi-domain psychiatric and healthy populations’ neuro-phenotyping [87]. It understands mental disease in terms of domain-wide inequities rather than focusing on a particular issue.

### 6.6. PREDICT

It is a massive dataset with EEG data open to the public [82]. A lot of data repositories hold patient-specific information, as well as house imaging. The user can only find patient-specific EEG data in a few places, including the Patient Archive for EEG Data and Analytical Instrument. It uses EEG data storage, tasks, and computational algorithms to categorize neuropsychiatric and neurological patients and give them a structured platform. Patient repository for EEG data + computational tools (PRED+CT) incorporates EEG data based on patient or normal side effect scores, gender, and age using the MATLAB toolbox. https://www.mathworks.com/products/matlab.html accessed on 27 November 2024

## 7. Future Research Directions

Integrating AI and EEG in depression detection has shown promising results, yet it also presents many future directions and challenges that warrant exploration. This study addresses several key areas and challenges for future research. Addressing these challenges will be essential for translating AI-based EEG depression detection from research settings to practical, real-world applications, ultimately improving diagnostic accuracy and patient outcomes.

### 7.1. Enhancing Depression Diagnosis via Data Augmentation

Numerous data augmentation techniques, such as GANs, can produce synthetic data to expand the datasets available for EEG-based depression diagnosis. Collaborative projects like RDoC, STAR*D [88], and IMAGEN aim to collect extensive and diverse datasets from multiple sites to optimize the translatability of research findings to real-world settings. Such projects should also be explored for EEG data, as seen in the iSPOT-D and CAN-BIND-I studies. The development of wireless and portable EEG equipment can also aid in the early detection and timely intervention of disorders.

The EEG data analysis could be enhanced by combining it with additional techniques like functional near-infrared spectroscopy (fNIRS) or functional magnetic resonance imaging (fMRI) [57]. Furthermore, researching vast datasets of people of all ages and genders can enhance the performance of classification studies. This can help provide the best care possible to people of all ages and genders, including children, seniors, females, and males. Various sources can be used to collect data, including students, IT experts, and working women. Finally, datasets with information on various forms of depression should be studied [41].

### 7.2. Exploring Feature Extraction Techniques for Automated Depression Diagnosis

To determine the scale of depression severity it is necessary to explore different feature categories, and for automated depression diagnosis, investigating the interplay between various feature extraction techniques and various pre-trained networks, such as DenseNet, VGG16, GoogleNet, ResNet, etc., is crucial. It is highly desirable to have methods for analyzing complicated, non-linear, and non-stationary EEG signals. Numerous characteristics can be investigated, including inter-hemispheric asymmetry in numerous sub-bands, including theta, delta, alpha, and beta [59]. A rising number of non-linear analysis approaches, including higher-order spectra characteristics, fuzzy entropy, and RQA, are being investigated for use in the diagnosis of depression. A combination of spatial features and amplitude-range differences in alpha rhythm can also be used as inputs for depression detection across various classifiers [56].

### 7.3. Optimizing EEG Channel Selection for Accurate Depression Diagnosis

The selection of the appropriate number of EEG channels poses a significant obstacle to deploying the model in portable clinical settings [89]. The ideal number of electrodes needed to accurately diagnose depression has to be looked into. To address this issue, efficient methods for feature selection can be explored, such as the differential evolution method and a robust optimization algorithm [90]. Its crossover and mutation operations can be further investigated to improve its performance. The method can be used to optimize a variety of features.

### 7.4. Exploring Transfer Learning and Encoder-Decoder Architectures Using Deep Neural Networks

There is growing interest in evaluating the performance of previously trained transfer learning models, including ResidualNet, GoogleNet, DenseNet, AlexNet, VGG-16, and others, to create a framework for diagnosing depression [91]. Additionally, it is possible to explore encoder-decoder architectures, combining the CNN with the RNN to create fully automated report generation for depressed patients. This can aid in the development of explainable AI models. Although there has been some research on deep neural networks for depression diagnosis, this has not yet been investigated using algorithms like the DBN, PNN, and ELM. Finally, one can assess the effectiveness of a fuzzy-based classification technique for diagnosing depression [22].

### 7.5. Integrating IoT and EEG for Remote Patient Monitoring

The suggested models should be integrated with the IoT infrastructure for remote patient monitoring. The development of mobile applications that can evaluate the severity of depression in patients without requiring doctor supervision is possible [5].

### 7.6. Challenges and Opportunities in EEG Analysis of Depression

The analysis and interpretation of EEG data concerning depression, particularly in tasks like music perception, is still a challenging area. There are opportunities for further research to explore the impact of mental disorders on brain oscillations in more natural situations, especially in cases of major depression. EEG source localization techniques can be employed to examine alterations in the brains of individuals with depression [57].

### 7.7. Distinguishing Between Depression Types

Finding biomarkers to distinguish between episodes of major depressive disorder and remission is difficult. For this, non-linear biomarkers based on EEG and the degree of physiological complexity corresponding to the severity of depression are being studied [79]. It might be challenging to correctly identify bipolar depression because it is frequently misinterpreted as unipolar depression [80]. The various bipolar depressive disorder (BDD) phases have been distinguished using smartwatches and self-reports. However, no one ML model worked well for all patients. Data-driven methods based on EEG may provide a solution to this issue.

## 8. Conclusions

The integration of EEG data with AI models represents a significant advancement in the diagnosis and treatment of depression, combining EEG’s high temporal resolution, non-invasiveness, and cost-effectiveness with the analytical power of AI. This survey provides a comprehensive review of state-of-the-art methods and future directions in diagnosing depression using EEG signals, highlighting progress and identifying key challenges. Our review spans the entire depression diagnosis pipeline, from EEG data acquisition and preprocessing to feature extraction and selection, followed by detailed discussions on classification methods. We compare the effectiveness of ML and advanced DL approaches in detecting depression, providing insights into their respective strengths and limitations. The survey examines diverse datasets used in depression research, including the HBN, EMBARC, Depresjon, Transdiagnostic Cohorts, MIPDB, and PREDICT. These datasets are vital for developing and validating robust diagnostic models. The review underscores how recent research demonstrates that both linear and non-linear features of EEG signals when processed through advanced ML and DL techniques can achieve high accuracy in identifying depressive states. The main objective of this study is to demonstrate the effectiveness of computer-aided methods for diagnosing depression and predicting treatment outcomes. In discussing the background and context of depression detection, we emphasize the primary methods—DL frameworks using neural networks and ML methods—that have garnered the most research attention. Looking ahead, several areas for future research are identified. Enhancing depression diagnosis with data augmentation techniques can improve the robustness of the models. Optimizing EEG channel selection and exploring transfer learning and encoder-decoder architectures using deep neural networks present opportunities to leverage pre-trained models and improve diagnostic accuracy. Investigating advanced feature extraction techniques for automated depression diagnosis can further enhance the performance of ML and DL models. Integrating IoT devices with EEG for remote patient monitoring can facilitate continuous and real-time mental health assessment. Finally, distinguishing between different types of depression remains a critical area for research. This survey serves as an organized and valuable reference for researchers working on depression detection using EEG signals, providing insights and guidance for future advancements in the field.

## Figures and Tables

**Table 1:** The analysis of the approaches based on the frequency.

No.	Method Type	Method Frequency%
1	Traditional-based approach	57%
2	Deep learning-based approach	43%

**Table 2:** Examining the most commonly used sub-techniques through a frequency-based analysis.

No.	Method Type	SVM	KNN	LR	CNN	ANN	LSTM
1	Traditional-based approach	46%	36%	18%	-	-	-
2	Deep learning-based approach	-	-	-	49%	8%	43%

**Table 3:** The used databases for academic purposes.

Academic Database	Link
IEEEXplore	https://ieeexplore.ieee.org/ (accessed on 27 November 2024)
ScienceDirect	http://www.sciencedirect.com/ (accessed on 27 November 2024)
Springerlink	https://link.springer.com/ (accessed on 27 November 2024)
Elsevier	https://www.elsevier.com (accessed on 27 November 2024)
American Psychological Association (APA)	http://www.https://www.apa.org/ (accessed on 27 November 2024)
Wiley	https://www.wiley.com (accessed on 27 November 2024)

**Table 4:** The standards for selecting or rejecting articles.

Inclusion Criteria	Exclusion Criteria
The review focuses on depression detection using EEG data	Articles that use other modalities such as social media analysis were excluded
Depression detection methods based on two categories (machine learning and deep learning) were considered	Other categories of depression detection methods, such as model-based approaches, were not considered
For inclusion, only publications written in English were considered	Publications in languages other than English were not recognized
Only papers published between 2016 and 2024	Papers were not indexed in ISI
Publications adhered to the rules of the citation number	Papers did not meet the minimum requirements of the citation

## Data Availability

No new data were created or analyzed in this study.
